# The influence of different diets on metabolism and atherosclerosis processes—A porcine model: Blood serum, urine and tissues ^1^H NMR metabolomics targeted analysis

**DOI:** 10.1371/journal.pone.0184798

**Published:** 2017-10-09

**Authors:** Adam Zabek, Robert Paslawski, Urszula Paslawska, Wojciech Wojtowicz, Katarzyna Drozdz, Sergio Polakof, Marzena Podhorska, Piotr Dziegiel, Piotr Mlynarz, Andrzej Szuba

**Affiliations:** 1 Bioorganic Chemistry Group, Department of Chemistry, Wroclaw University of Technology, Wyb. Wspianskiego, Wroclaw, Poland; 2 WROVASC—Regional Specialist Hospital in Wroclaw, Research and Development Centre, Kamienskiego, Wroclaw, Poland; 3 Department and Clinic of Internal and Occupational Diseases and Hypertension Wroclaw Medical University, Borowska, Wroclaw, Poland; 4 Department of Internal Diseases with Clinic for Horses, Dogs and Cats, Faculty of Veterinary Medicine, Wroclaw University of Environmental and Life Sciences, Norwida, Wroclaw, Poland; 5 Division of Angiology, Wroclaw Medical University, Pasteura, Wroclaw, Poland; 6 4th Military Hospital in Wrocław, Weigla, Wrocław, Poland; 7 Clermont Université, Université d'Auvergne, Unité de Nutrition Humaine, BP 10448, Clermont-Ferrand, France; 8 INRA, UMR 1019, UNH, CRNH Auvergne, Clermont-Ferrand, France; 9 Department of Histology and Embriology, Wroclaw Medical University, Wroclaw, Poland; University of Texas at Austin, UNITED STATES

## Abstract

The global epidemic of cardiovascular diseases leads to increased morbidity and mortality caused mainly by myocardial infarction and stroke. Atherosclerosis is the major pathological process behind this epidemic. We designed a novel model of atherosclerosis in swine. Briefly, the first group (11 pigs) received normal pig feed (balanced diet group—BDG) for 12 months, the second group (9 pigs) was fed a Western high-calorie diet (unbalanced diet group—UDG) for 12 months, the third group (8 pigs) received a Western type high-calorie diet for 9 months later replaced by a normal diet for 3 months (regression group—RG). Clinical measurements included zoometric data, arterial blood pressure, heart rate and ultrasonographic evaluation of femoral arteries. Then, the animals were sacrificed and the blood serum, urine and skeletal muscle tissue were collected and ^1^H NMR based metabolomics studies with the application of fingerprinting PLS-DA and univariate analysis were done. Our results have shown that the molecular disturbances might overlap with other diseases such as onset of diabetes, sleep apnea and other obesity accompanied diseases. Moreover, we revealed that once initiated, molecular changes did not return to homeostatic equilibrium, at least for the duration of this experiment.

## Introduction

Overconsumption of highly processed foods is considered a key factor in the development of many civilization-induced health disorders [1]. Overconsumption of food rich in animal fat and sugars together with a Western lifestyle and certain environmental factors lead to profound metabolic changes. Determining the molecular background of early diet-induced atherosclerosis may help find new biomarkers for an early diagnosis of civilization diseases and drugs to treat them. Next to genomics and proteomics, metabolomics might be the method of choice for molecular diagnostics of atherosclerosis. It analyses low molecular weight compounds and allows tracking of metabolite flux changes between biochemical pathways and enzyme activities [2].

Among others, risk factors for atherosclerosis include an unbalanced diet leading to obesity and related disorders like hypertension, insulin resistance and diabetes [3]. Atherosclerosis, obesity and other civilization related disorders are currently investigated by holistic metabolomic tools using mass spectrometry (MS) hyphenated with separation methods as well as by nuclear magnetic resonance (NMR) [4,5]. These studies are conducted on groups of patients or animal models [6,7]. Many rodent and non-rodent species have served as models in translational studies. However, the pathomechanisms of numerous diseases in those species do not always reflect the pathomechanisms of human diseases. Hence, it is important to choose animals such as pigs or small ruminants, which are closer anatomically and physiologically to humans, and can be well tailored to scientific demands [8,9]. Pigs are often used as models in studies on atherosclerosis and diseases accompanying obesity due to the biological similarities shared by pigs and humans [10–14]. The porcine model for obesity has been explored using metabolomics [15,16], however we could not find studies on metabolomic profiling of atherosclerotic processes occurring in swine.

Due to the fact that pigs have a similar cardiovascular system, a similar metabolism, tendency to overconsumption and develop spontaneous atherosclerosis [17], we have chosen to carry out our study using this species. The study was designed to determine biomarkers of atherosclerosis, to test whether a normal body metabolism in pigs can be restored by adjusting the diet, and to find out what effect a diet change has on atherosclerosis. For this purpose, 28 pigs were divided into three groups. The first group (11 pigs) received normal pig feed (balanced diet group—BDG), the second group (9 pigs) was fed a Western high-calorie diet (unbalanced diet group—UDG) and the third group (8 pigs) received a Western type high-calorie diet for nine months which was later replaced by a normal diet (regression group—RG). The metabolomics studies were performed on blood serum, urine and tissue samples.

## Materials and methods

### Ethics statement

The studies were performed with the approval No. 23/2009 of the 1^st^ Local Ethics Committee for Animal Experimentation of the Institute of Immunology and Experimental Therapy in Wroclaw, Poland. All animals received humane care in compliance with the 8^th^ edition of the *Guide for the Care and Use of Laboratory Animals* published by the National Institute of Health (http://www.ncbi.nlm.nih.gov/books/NBK54050/).

### Animals

The study included 28 Polish Landrace female pigs (white domestic pig). The inclusion criteria were as follows: body weight of 40 kg, a normal clinical examination, normal blood cell morphology values (haematocrit, plasma haemoglobin levels), and normal concentrations of serum creatinine, urea, total protein, albumin and electrolytes. Our research stands out for long duration, which prompted us to choose females to form the experimental group. Males after puberty became aggressive to each other. We chose females to avoid redundant stress and fighting for dominance.

### Experimental set up and sample collection

All the pigs were housed in a single room, divided into three pens (one pen per group), with a temperature of 18–20°C and 60–75% humidity. All the animals had unlimited access to water. The control group (balanced, low fat diet), which consisted of 11 pigs, was fed for 12 months a standard, commercial, balanced feed specifically prepared for the nutritional needs according to Feeding Standards for Pigs. Pigs over 60 kg were fed a feed for adult sows with a total energetic value of 2100 kcal/kg that contained: 14.7% protein, 3.1% fat, 4.7% crude protein, 90.44% dry mass, 6.06% ash, 0.5% NaCl, 1.05% Ca, 0.77% P, 0.62% lysine, 0.24% methionine, 0.3% cystine, 0.48% threonine, 0.183% tryptophan, 13243 IU/kg vit. A, 2000 IU/kg vit. D_3_, 81.65 mg/kg vit. E, 4.11 mg/kg vit. B_1_, 7.16 vit. B_2_, 50.22 mg/kg vit. PP, 24.29 mg/kg vit. B_5_, 6.11 mg/kg vit. B_6_ and 36 μg/kg vit. B_12_. This feed had a low monohydrate content. The control group had limited access to food, which was proportional to body mass, with a maximum calorie intake of 4200 kcal/pig/day.

The experimental diet (unbalanced, high fat), had a 3200 kcal/kg energy content. Nine pigs were fed this diet, which had a 5x higher fat level (obtained by adding tallow), 13% higher protein level and a 4% addition of saccharose compared to the standard diet. Pigs in this group had unlimited access to food and consumed 4kg/pig/day during the period of highest food intake. The third (the high fat/low fat) group, consisting of eight pigs, was fed an unbalanced diet for nine months and then a standard, balanced diet for three months. All diet ingredients are included in supplementary data information (S1 Text).

Blood was drawn twice–at the beginning and at the end of the study. The complete blood count (CBC) was analyzed using an ABC-Vet analyzer, while the biochemical analyses were performed using the MaxMat Pl analyser. The clinical data is summarized in Table 1. In addition we have checked glucose and insulin levels and we have not found any significant differences between the groups.

**Table 1:** Clinical laboratory data in the three study groups at the start and at the end (12 month) of the study.

	Balanced diet group		Unbalanced diet group		Regression group	
	Start	end	start	End	Start	end
Body weight (kg)	40	246±27.5	40	260±21	40	245±16.5
Lumbar subcutaneous fat (mm)	3.87±0.08	13.6±3	3.88±1	14.6±2.5	3.9±0.1	12±2.5
Neck subcutaneous fat (cm)	2.38±0.08	12.5±2.5	2.4±0.07	16±1.6*	2.39±0.06	12.4±2
Shoulder subcutaneous fat (cm)	2.34±0.08	11.6±2.4	2.4±0.07	14.4±1.8*	2.4±0.1	11.4±2
Triglycerides, TG (mmol/l)	-	0.32±0.2	-	0.53±0.34	-	0.49±0.21*
Total Cholesterol, TC (mmol/l)	-	2.1±0.45	-	2.02±0.48	-	1.9±0.51
HDL-C (mmol/l)	-	0.75±0.13	-	0.88±0.34	-	0.65±0.1*
LDL-C (mmol/l)	-	1.09±0.24		1.17±0.17	-	1.18±0.2
Glucose (mg/dl)	87,7±14,42	94,6±13,7	96,8±18,7	102,5±27,4	100,3±22	103,8±26,1
Insulin (μg/ml)	38,8±4,78	45,7±14,65	42,3±11,6	52,8±19,7	53,3±30	79,4±52,9

* p < 0.05

The animals were sacrificed at the end of the study and tissue sample including subcutaneous fat and aorta were obtained for further analysis. Basic histopathological analysis included haematoxylin and eosin staining of the arterial rings from the same site on the femoral artery of all the animals and measurements of intima, media and adventitia thickness. Thickness of intima was chosen as the histological marker of atherosclerosis and compared with ultrasonographic measurements of the intima-media complex.

Clinical evaluation of animals was performed every 3 months. Clinical measurements included zoometric data, arterial blood pressure and heart rate, ultrasonographic evaluation of arteries for measurements of intima-media thickness (Table 2). The ultrasonographic measurements of subcutaneous fat were performed by a single researcher using the Aloka 4000+ scanner (Aloka Company, Japan) equipped with a 5–10 MHz linear transducer. The transducer was placed at three sites: 1) on the back–approximately 6 cm to the left of the spine at the perpendicular line demarcated from the last rib to the vertebral column, 2) on the neck—3 cm from the dorsa midline at the elbow line 3) on the shoulder—8 cm from the dorsal midline at the elbow line. These are the standard sites of fat accumulation in breeding pigs [15].

**Table 2:** Ultrasound measured Intima-Media complex thickness (IMT) and the mean thickness of intima, media and adventitia obtained from a histological analysis of femoral arteries at the same location as ultrasound measurements in all study groups.

	IMT—ultrasound (μm) mean±SD	Intima (μm) mean±SD	Media (μm) mean±SD	Adventitia (μm) mean±SD
**BDG**	638 ± 87	85 ± 66	553 ± 55	254 ± 49
**UDG**	705 ± 108*	143 ± 87*	562 ± 79	269 ± 77
**RG**	686 ± 102	141 ± 90	545 ± 73	287 ± 49

* p<0.05 for BDG vs. UDG

### Sample preparation for proton NMR spectroscopy

Serum and urine were sampled from the animals. Serum was sampled from the peripheral vein and centrifuged for 10 minutes at 4 000 x g. All the samples were frozen in liquid nitrogen immediately after collection and stored at—80°C until analysis.

Prior to the metabolomic experiment, the collected serum samples were thawed at room temperature and vortexed. Next, the mixtures of 200 μL of serum and 400 μL of saline solution (prepared from 0.9% NaCl, 15% D_2_O and 3 mM TSP (trimethylsilyl-2,2,3,3-tetradeuteropropionic acid sodium salt)) were mixed. After centrifugation (12 000 x g, 10 min), an aliquot of 550 μL of the sample, the supernatant was transferred to a 5 mm NMR tube. Samples were kept at 4°C prior to measurement.

Urine samples were obtained through urinocentesis of the urinary bladder using a 0.9ø standard needle and a 10ml sterile syringe. All urine samples were thawed at room temperature and mixed using a vortex mixer. The samples were centrifuged for 10 minutes at 12 000 x g. 400 μL of the supernatant was then transferred into a new Eppendorf tube. The samples were mixed with 200 μL of PBS (0.5 M, pH = 7.00, 33% D_2_O, 3 mM NaN_3_ and 3 mM TSP). The samples were mixed again and an aliquot of 550 μL was transferred into a 5 mm NMR tube.

Prior to the NMR measurements, muscle tissue fragments were weighed and frozen in liquid nitrogen for at least 10 minutes. The tissues were then transferred into a mechanic steel bead homogenizer (TissueLyser LT; QIAGEN, Hilden, Germany). During the first step of homogenization, frozen tissue samples were disrupted by 1.2 mL of a methanol:chloroform (2:1 ratio) mixture for 10 minutes at a frequency of 50 Hz. The obtained solution was supplemented with 400 μL chloroform and homogenized again. To remove any cellular debris or insoluble material, the samples were centrifuged at 1 000 x g for 15 minutes. The 1 mL aliquots of supernatant of aqueous tissue extract were transferred into new Eppendorf tubes and evaporated using the speedvac concentrator. Finally, the pellets were suspended in 500 μL of a 0.5 M phosphate buffer solution (PBS), (pH = 7.4; 10% D_2_O; 3 mM sodium azide; 1 mM trimethylsilyl-2,2,3,3-tetradeuteropropionic acid sodium salt (TSP) as an internal standard) and transferred into 5 mm NMR tubes and stored at 4°C until analysis.

### ^1^H NMR measurements

The NMR spectra of serum, urine and tissue samples were recorded at 300 K using the Avance II spectrometer (Bruker, GmBH, Germany) operating at a 600.58 MHz proton frequency. The NMR spectra of serum were recorded using a CPMG pulse sequence with water presaturation in a Bruker notation. For each sample, 128 subsequent scans were collected with a 400 μs spin-echo delay; 80 loops; 3.5 s relaxation delay; acquisition time of 2.73 s; TD of 64k; SW of 20.01 ppm.

The NMR spectra of urine and tissue were recorded with the use of the nuclear Overhauser effect spectroscopy, NOESY pulse sequence with water presaturation in a Bruker notation: with a relaxation delay of 3.5 s; acquisition time of 1.36 s; 128 transients; TD of 64k; SW of 20.01 ppm.

The spectra were processed using 0.3 Hz of line broadening and were manually phased and baseline corrected using Topspin 1.3 software (Bruker, GmBH, Germany) and referenced to α-glucose signal δ = 5.225 ppm for serum samples and to the TSP resonance at δ = 0.000 ppm for the urine and tissue samples. The correlation optimized warping algorithm, COW, and the *i*coshift algorithm implemented in Matlab (Matlab v. 8.1, Mathworks Inc.) were used to correct the peak positions (alignment). The spectra were normalized using the Probabilistic Quotient Normalization (PQN) method.

### Preprocessing of variables prior to analysis

A total of 27 serum, 19 urine and 26 tissue metabolites were assigned. The concentration of any NMR measured metabolite was obtained as a signal integral of the non-overlapping resonances (or a cluster of partly overlapping resonances). The metabolite resonances were identified according to assignments to chemical shifts published in the literature and online databases (Biological Magnetic Resonance Data Bank and Human Metabolome Database). All variables (originating from various fluids) were scaled to unit standard deviation, which guaranteed their equal importance (equal weight) in the model construction.

### Multivariate data analysis for biomarker identification

The discriminant version of the Partial Least Squares regression (PLS-DA) was adopted with a 7-fold cross validation procedure (CV, 1/7 of the samples being excluded from calculations in each round) to determine the variation between data sets. Variable selection using the VIP-plots with the jackknife confidence interval and confidence level at 0.95 was conducted to improve the obtained models. The variables with VIP values below 0.8 and a jackknife confidence interval overlapping 0 from the following analysis were removed. The new models were re-built on the basis of selected variables. This approach was repeated to select variables exceeding 0.8 and without overlapping 0 by the jackknife confidence interval.

### Fusion data

Relative integral of resonances obtained from paired serum, urine, tissue and medical data samples were fused into one data matrix. For the purposes of model calculation, names of metabolites identified in the serum, urine and tissue have been replaced with X_S, X_U and X_T structure, respectively, where X was the name of metabolite. The clinical laboratory data (Table 1) were left without any markings.

The prediction performance of the PLS-DA models was estimated based on receiver operating characteristic (ROC) curves and area under curve (AUC) values. For this purpose, a *perfcurve* function from the Matlab statistical tool-box (Matlab v. 8.1, Mathworks, Inc.) was adopted. Specificity and sensitivity were determined according to sample class prediction using the 7-fold cross-validated predicted values of the fitted *Y*-predcv (implemented in SIMCA-P+ software) for observations in the model.

### Statistical data analysis

The percentage difference (PD) and relative standard deviation (RSD) were calculated using STATISTICA 10 for each serum, urine and tissue metabolites. The percentage difference was calculated based on the mean values of relative signal integrals in each group. The calculations were carried out from left to right. The statistical significance based on the Student t test was calculated for the chosen metabolites.

## Results

**Fig 1:**
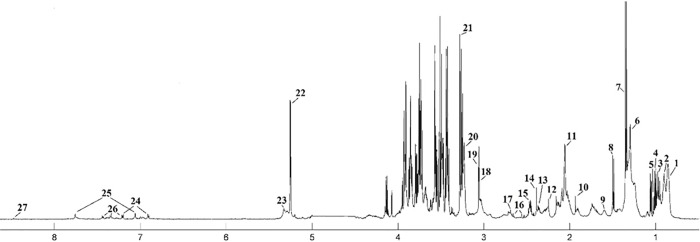
The median spectrum ^1^H NMR obtained from serum samples in the balanced diet group. The following metabolites are identified: 1, Lipid (L1): LDL CH_3_–(CH_2_)*n*–; 2, Lipid (L2): VLDL CH_3_– (CH_2_)*n*–; 3, Leucine; 4, Valine; 5, Isoleucine; 6, Lipid (L3): LDL/VLDL CH_3_– (CH_2_)*n*–; 7, Lactate; 8, Alanine; 9, Lipid (L4): VLDL CH_2_–CH_2_–C = O; 10, Acetate; 11, *N*-acetylated glycoproteins; 12, Acetone; 13, Glutamate; 14, Pyruvate; 15, Glutamine; 16, Citrate; 17, Dimethylamine (DMA); 18, Creatine; 19, Creatinine; 20, Choline (Chol) + Phosphocholine (PC) + glycerophosphocholine (GPC); 21, Betaine; 22, Glucose; 23, Lipid (L5): unsaturated lipids–CH = CH–; 24, Tyrosine; 26, Histidine; 27, Phenylalanine; 28, Formate.

**Fig 2:**
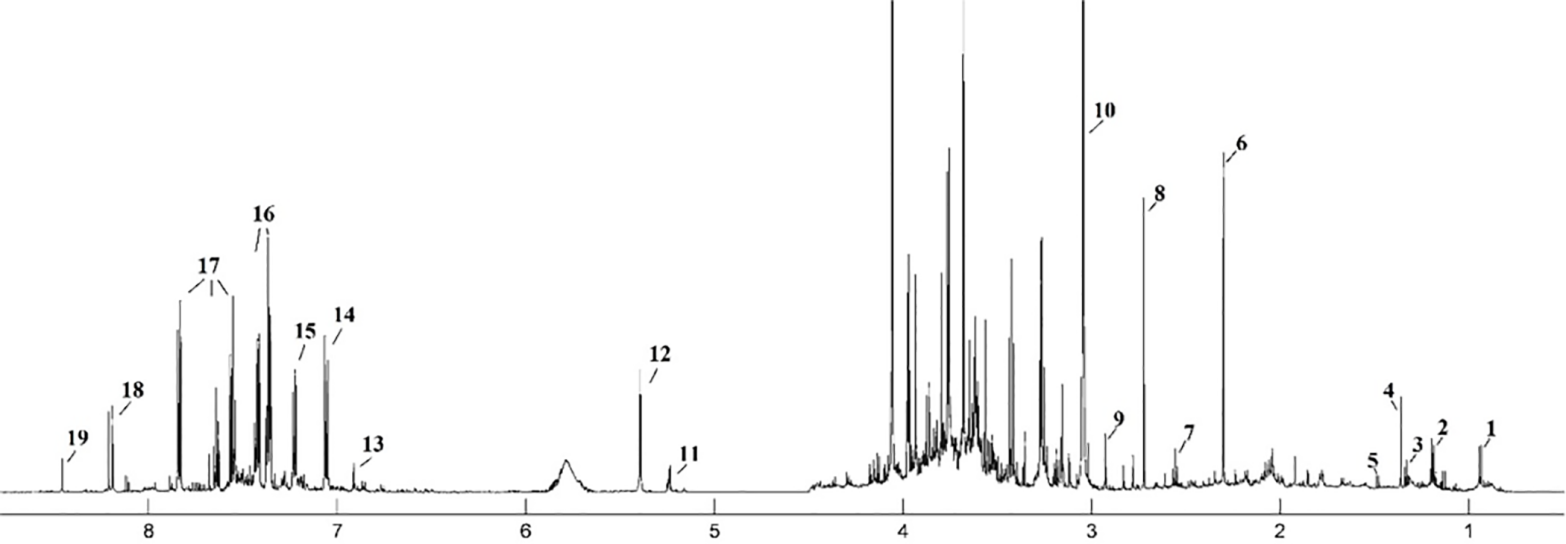
The median spectrum ^1^H NMR obtained from urine samples in the balanced diet group. The following metabolites are identified: 1, Isovalerate; 2, Isopropanol; 3, Threonine; 4, 2-Hydroxyisobutyrate; 5, Alanine; 6, Unk_1 δ 2.3 ppm; 7, β-Alanine 8, Dimethylamine (DMA); 9, *N*,*N*-Dimethylglycine; 10, Creatinine; 11, Unk_2 δ 5.23 ppm; 12, Allantoin; 13, Unk_3 δ 6.91 ppm; 14, Unk_4 δ 7.02 ppm; 15, Unk_5 δ 7.22 ppm; 16, *N*-Phenylacetylglutamine; 17, Hippurate; 18, Unk_6 δ 8.22 ppm; 19, Formate.

**Fig 3:**
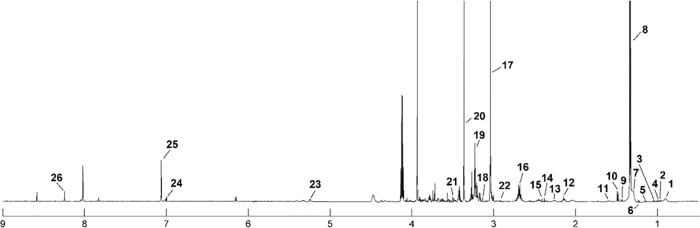
The median spectrum ^1^H NMR obtained from tissue samples in the balanced diet group. The following metabolites are identified: 1, L1; 2, Leucine; 3, Valine; 4, Isoleucine; 5, Unk_1; 6, Unk_2; 7, L2; 8, Lactate; 9, Unk_3; 10, Alanine; 11, L3; 12, Methionine; 13, L4; 14, Pyruvate; 15, Succinate; 16, Carnosine; 17, Creatine; 18, Malonate; 19, Betaine; 20, Methanol; 21, Glycine; 22, *N*,*N*-Dimethylglycine; 23, Glucose; 24, Histidine; 25, Methylhistidine; 26, IMP.

The median ^1^H NMR spectrum of serum with 27 metabolites, the urine spectrum of 19 and tissue spectrum of 26 assigned metabolites in the balanced diet group is presented as an example in Figs 1, 2 and 3. The metabolite resonances signals were identified according to assignments published in the literature and in on-line databases (http://hmdb.ca).

The statistical calculation almost coincides with the VIP plot for serum metabolites in each comparison (S1 Table). The BDG vs. RG is accompanied by changes in the level of leucine, valine, isoleucine, alanine and acetone, RG vs. UDG in alanine and betaine perturbation, while leucine, valine, isoleucine, alanine acetone, pyruvate and betaine can be used to differentiate the BDG and UDG groups. Among these metabolites two, alanine and isoleucine, are important only in VIP selection. (Table 3, highlighted metabolites; S1 Fig).

**Table 3:** Changes in the level of statistically significant* and VIP selected serum metabolites. The listed metabolites are important at least in one comparison.

Metabolite	Percentage difference			Relative Standard Deviation [%]		
	BDG vs. RG	RG vs. UDG	BDG vs. UDG	BDG	RG	UDG
Leucine	-22.5*	2.4	-20.2*	11.1	8.7	8.8
Valine	-24.0*	3.6	-20.5*	15.3	11.5	11.3
Isoleucine	-19.6*	8.7	-11.0	11.6	12.1	11.6
Alanine	28.3*	-19.1*	9.3	14.7	21.9	14.3
Acetone	-29.3*	-17.7	-46.4*	17.4	25.5	29.3
Pyruvate	26.0	-7.0	19.0*	15.0	36.2	20.4
Betaine	-2.1	32.0*	30.0*	17.6	17.7	19.0

*(*p* value < 0.05 for Student t test)

Similarly, the statistical analysis of urine samples is convergent with the VIP plot (S1 Table). It revealed that six metabolites were important (Unk 1, *N*,*N*-dimethylglycine, creatinine, Unk 4, Unk 5) for BDG vs. RG comparison, four for RG vs. UDG (DMA, *N*,*N-*dimethylglycine, creatinine, Unk 6) and six (Unk, 1, Unk 4, Unk 5, Unk 6, *N-*phenylacetylglutamine, hippurate) for BDG and UDG, respectively (Table 4, highlighted metabolites; S2 Fig). Some metabolites were significant only in one test passing VIP selection namely *N*,*N*-dimethylglycine and creatinine in BDG vs. RG comparison and hippurate for BDG vs. UDG relation.

**Table 4:** Changes in the level of statistically significant* and VIP selected urine metabolites. The listed metabolites are important at least in one comparison.

Metabolite	Percentage difference			Relative Standard Deviation [%]		
	BDG vs. RG	RG vs. UDG	BDG vs. UDG	BDG	RG	UDG
Unk 1 δ 2.3	55.6*	-1.8	54.0*	41.8	41.8	16.9
DMA	41.1	-21.5*	20.0	61.4	61.4	13.6
*N*,*N*-Dimethylglycine	51.9	-47.3*	4.8	63.9	63.9	22.7
Creatinine	48.4	-24.8*	24.4	70.2	70.2	20.1
Unk 4 δ 7.02	60.8*	-2.5	58.5*	42.6	42.6	24.3
Unk 5 δ 7.22	60.8*	-2.5	58.5*	35.2	35.2	24.4
*N*-Phenylacetylglutamine	73.5	-8.2	66.3*	49.2	49.2	19.3
Hippurate	45.0	26.8	69.7	78.9	78.9	35.5
Unk 6 δ 8.22	-6.2	91.8*	86.9*	66.6	66.6	111.7

* (*p* value < 0.05 for Student t test)

The statistical and VIP plot analysis of the tissue samples revealed five (L1, leucine, valine, L3, L4), ten (L1, leucine, valine, isoleucine, lactate, L3, methionine, L4, creatine, methylhistidine) and seven (leucine, lactate, methionine, creatine, glucose methylhistidine, IMP) metabolites, which were found to be important in distinguishing between particular diets BDG vs. RG, RG vs. UDG as well as BDG vs. UDG, respectively (Table 5, highlighted metabolites; S3 Fig, S1 Table).

**Table 5:** Changes in the level of statistically significant* and VIP selected tissue metabolites. The listed metabolites are important at least in one comparison.

Metabolite	Percentage difference			Relative Standard Deviation [%]		
	BDG vs. RG	RG vs. UDG	BDG vs. UDG	BDG	RG	UDG
L1	-114.0*	57.9	-67.3	76.8	67.1	64.1
Leucine	-135.0*	74.2*	-81.2*	94.2	45.8	38.9
Valine	-202.3*	125.3*	-210.1	-6032.5	76.6	235.4
Isoleucine	151.0	-149.0	4.6	-47.1	-656.7	-104.7
Lactate	-20.6	-5.9	-26.4*	13.7	13.8	19.1
L3	-98.3*	47.5	-57.6	53.5	55.3	49.2
Methionine	-56.9	-2.9	-59.5*	29.9	25.2	32.1
L4	-118.3*	63.0	-68.0	96.1	60.4	72.2
Creatine	-27.6	5.3	-22.4*	11.1	16.5	19.8
Glucose	-48.4	14.1	-34.9*	21.8	54.8	17.2
Methylhistidine	-30.3	-14.1	-44.0*	21.9	15.3	28.6
IMP	-41.9	8.1	-34.1*	17.4	54.1	14.3

* (*p* value < 0.05 for Student t test)

### Fusion data analysis

**Fig 4:**
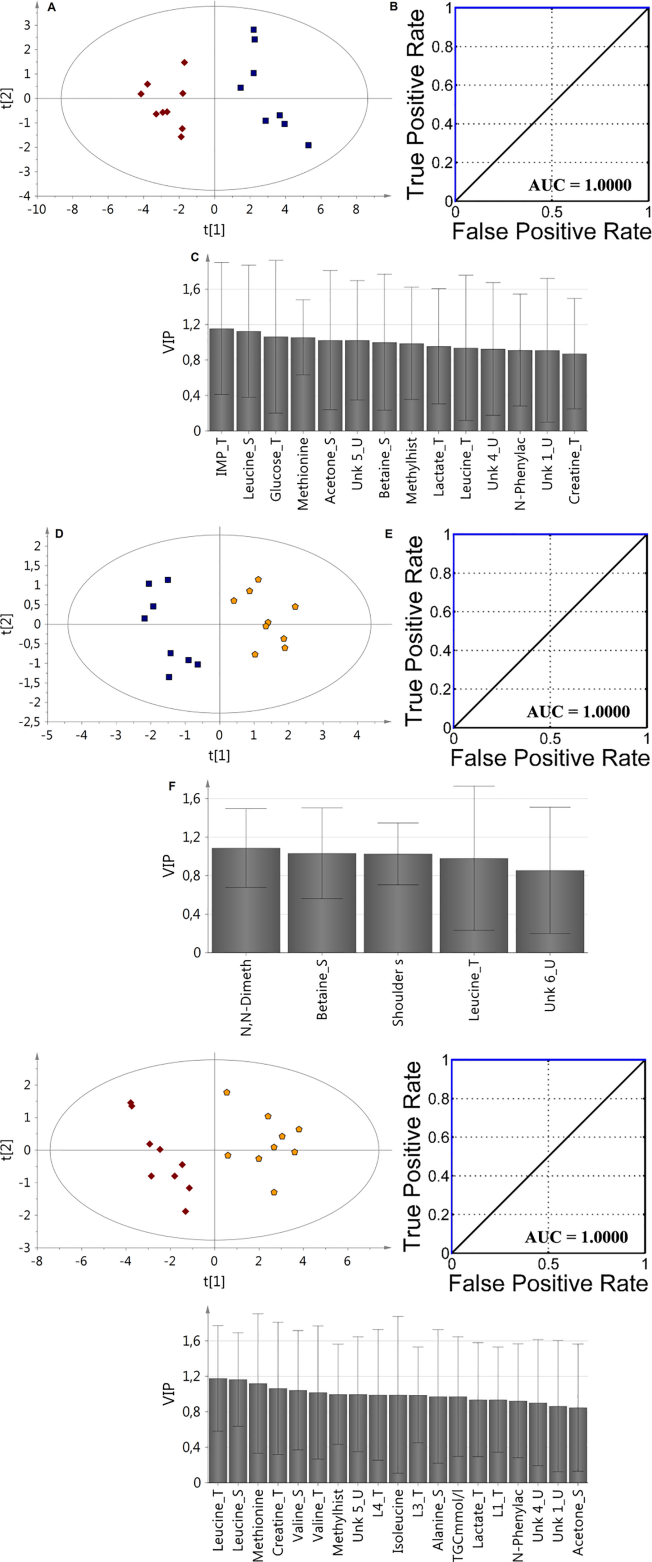
The VIP-PLS-DA model ROC curve and AUC values obtained for fusion data samples based on selected variables according to the VIP plots with the jackknife confidence interval: A,B,C (BDG vs. RG); D,E,F (RG vs. UDG); G, H, I (BDG vs. UDG). Red diamonds—balanced diet group (BDG); blue boxes—regression group (RG); yellow pentagons—unbalanced diet group (UDG).

For improving model parameters the VIP-PLS-DA approach for fusion data was applied including two type of parameters, namely from all biological media metabolite levels and clinical data (Table 1, Fig 4). The data fusion based on the serum, urine, tissue and medical data definitely improved of the calculated model’s parameters (S2 Table). For each comparison, the AUC value was found to be 1. According to VIP plots analysis, the most important metabolites were selected for each model: BDG vs. RG: (IMP_T, Leu_S, glucose_T, Met_T, acetone_S, Unk_5_U, betaine_S, methylhistidine_T, lactate_T, Unk_4_U, *N*-phenylacetylglutamine_U, Unk_1_U, creatine_T), RG vs. UDG: (N, N-dimethylglycine_U, betaine_S, shoulder subcutaneous fat, Leu_T, Unk_6_U), BDG vs. UDG: (Leu_T, Leu_S, Met_T, creatine_T, Val_S, Val_T, methylhistidine_U, Unk_5_U, L4_T, Ile_S, L3_T, Ala_S, TGC, lactate_T, L1_T, *N*-phenylacetylglutamine_U, Unk_4_U, Unk_1_U, acetone_S).

## Discussion

Atherosclerosis is a result of complex interaction of various risk factors including high fat and carbohydrate–Western type diet, lack of physical activity and many others [18,19]. Although studied for decades, the molecular background of diet-induced atherosclerosis is not fully recognized [20].

In this study, paired samples of urine, serum and tissue obtained from three groups (BDG, RG and UDG) were investigated using univariate and multivariate analyses in relation to the obtained clinical data, ultrasound of Intima-Media complex thickness (IMT) measurements and histological analysis of femoral arteries. All these parameters well reflect the influence of diet on porcine metabolism. The atherosclerotic effect was proved by histopathological examination. In this study, we have shown the metabolic profiles in the early stage of atherosclerosis in the swine model with dietary changes and reversal.

The literature data concerning the role of branched chain amino acids (BCAAs) such as Leu, Ile and Val on the human organism is univocal [21–23]. These metabolites participate in glucose metabolism, protein synthesis [24–26], and affect the fat metabolism by regulating the secretion of leptin [27]. The high BCAAs level in obese patients was observed and positively correlated with body mass, insulin resistance [28], HOMA-IR (fasting insulin) [29] and glucose intolerance [30,31]. All these BCAA (Leu, Ile, Val) are negatively correlated with adiponectin, which regulates glucose level and fatty acids breakdown, positively with HOMA-IR, and selectively with C-peptide (Ile) and HbA1c (glycated haemoglobin) (Val, Leu) [32]. In our study, the increased levels of BCAA in serum were observed in group BDG vs. RG and group BDG vs. UDG, which indicates that a diet change does influence BCAA metabolism. However, the three months of regression diet might not be sufficient to reverse the metabolic changes in BCAA. The analysis of BCAA in tissue samples in the BDG vs. RG group revealed much lower BCAA levels in the BGD group accompanied by downregulation of Leu and Val and upregulation of Ile in the BGD. When comparing the BDG and UDG group, there was a statistically significant decrease in the concentration of Leu, while the concentration of Ile was at homeostatic level. The tissue comparison of the RG vs. UDG group revealed an increase in Leu and Val and a decrease in Ile, which indicates that after three months of a healthy diet the tissue metabolic changes are observed at the cell level.

Alanine metabolism is tightly associated with gluconeogenesis, which can be directly influenced by TCA cycle via pyruvate (which also decreases). In this study, the level of serum Ala was decreased in the RG and UDG groups compared to the BDG. The lowest value was observed in the RG group. However, the blood serum glucose level was almost constant and was not affected by diet type, while the content of tissue glucose increased for UDG (Table 5). Despite a reduced level of serum alanine and pyruvate, the TCA cycle seems to be intact, looking at citrate level. These findings show that despite 12 months of unbalanced diet the blood glucose homeostasis is maintained, which masks the metabolic disturbances already present in tissue, where the glucose level was increased in both UDG and RG (not statistically significant) diet group. The increase of tissue lactate can be associated with hypoxia condition [33] and we can also assume that these findings may be responsible for higher tissue glucose level for enriched diet. Also for these groups, the increased acetone level was observed, which most probably can be associated with accelerated fat metabolisms [34].

A decreased betaine serum level well corresponds with the published results [30]. This metabolite is known as an osmoregulator, methyl group donor and participates in the metabolism of fatty acids [35,36]. Moreover, its deficiency can lead to increased homocysteine levels, which is reported as a risk factor of atherosclerosis [37,38]. The tissue creatine level in both the RG (not statistically significant) and UDG groups was higher than in the control group (BDG). Creatine is the biochemical precursor of high energetic phosphocreatine and its higher storage may be associated with the applied diet. On the other hand, its higher level at the supplementation was found to be in negative correlation with IMP (known as the indicator of hypoxemia condition) during endurance exercise in humans [39, 40]. An increased level of methylhistidine in tissues may be a sign of muscle protein breakdown [41], while increased levels of tissue methionine may be a defense response against cell fat deposition [42]. Considering that the lipid tissue content in the RG and UDB diets was increased, we expected an increasing trend in the lipid profile, but no significant changes were found in the basic serum lipid profile (TC, LDL-C, HDL-C, TG). However, the methodology used in this study did not enable us to determine the full lipid profile. An interesting trend was also observed for inosine monophosphate, where IMP was found to be decreased in BDG group and at a similar level in RG and UDG, which can mean that at the cell level this metabolite is restored by changing the diet.

The urine metabolites reflect the metabolome and the gut microbiota activities and contents of the subject [43,44]. Among the array of metabolites, which are listed in Table 4, the levels of Unk 1 δ 2.3, DMA, *N*,*N*-dimethylglycine, creatinine, Unk 4 δ 7.02, Unk 5 δ 7.22, *N*-phenylacetylglutamine, hippurate, Unk 6 δ 8.22 are statistically significant. There was a decreased level of DMA in the RG vs. UDB signifying that the level of DMA was the lowest in the RG group and the highest in the BDG group. DMA is the product of choline metabolism by gut microbiota [45], but can also be derived from other sources such as metabolisms of TMAO [46] and asymmetric dimethylarginine (ADMA) as a product of dimethylarginine dimethylamino hydrolase (DDAH) action [47]. The decreased level of urine creatinine in RG and UDG vs. BDG group was accompanied by higher level of tissue creatine, which may indicate different protein turnover. *N-*Phenylacetylglutamine was found in plasma and urine of uremic subjects, while in the swine model is significantly downregulated in UDG group [48]. Hippurate was decreased in both RG and UDB groups in comparison to RDG. This finding is in accordance with the literature, where decreased hippurate levels were found in obese and insulin resistant Zucker (fa/fa) rats in comparison to lean Zucker rats (-/-) [49] and obese insulin resistant subjects to control group [50,51]. This decreased level of hippurate found in the UDB group may be associated with atherosclerosis, despite different gut microbiota in pigs and humans.

## Conclusion

Atherosclerosis results from complex metabolic disturbances, which are the result of an interplay between various risk factors including among others unbalanced diet, decreased physical activity leading to obesity, dyslipidemia, insulin resistance, diabetes, etc. Almost all these pathological conditions seem to overlap, showing similar changes in metabolite levels. However, metabolite level alterations may occur much earlier than clinical symptoms and changes in the markers investigated routinely, such as serum lipids or glucose and an increased thickness of the intima-media layer in carotid arteries.

## Supporting information

S1 Table The parameters of PLS-DA models obtained from ^1^H NMR analysis of serum, urine, tissue and fusion data samples. (DOC)

S2 Table The parameters of PLS-DA-VIP models obtained from ^1^H NMR analysis of serum, urine, tissue and fusion data samples. (DOC)

S1 Fig The VIP-PLS-DA models, ROC curve and AUC values obtained from serum samples based on selected variables according to the VIP plots with the jackknife confidence interval: A, B, C (BDG vs. RG); D, E, F (RG vs. UDG); G, H, I (BDG vs. UDG). Red diamonds—balanced diet group (BDG); blue boxes—regression group (RG); yellow pentagons—unbalanced diet group (UDG).
(DOC)

S2 Fig The VIP-PLS-DA model ROC curve and AUC values obtained from urine samples based on selected variables according to the VIP plots with the jackknife confidence interval: A,B,C (BDG vs. RG); D,E,F(RG vs. UDG); G,H,I (BDG vs. UDG). Red diamonds—balanced diet group (BDG); blue boxes—regression group (RG); yellow pentagons—unbalanced diet group (UDG).
(DOC)

S3 Fig The VIP-PLS-DA model ROC curve and AUC values obtained from tissue samples based on selected variables according to the VIP plots with the jackknife confidence interval: A,B,C (BDG vs. RG); D,E,F (RG vs. UDG); G, H, I (BDG vs. UDG). Red diamonds—balanced diet group (BDG); blue boxes—regression group (RG); yellow pentagons—unbalanced diet group (UDG).
(DOC)

S1 Text Information of the diet ingredients. (DOCX)
