# Supplementary material for: The influence of different diets on metabolism and atherosclerosis processes—A porcine model: Blood serum, urine and tissues 1H NMR metabolomics targeted analysis
Source: PLoS One. 2017 Oct 9;12(10):e0184798. doi: 10.1371/journal.pone.0184798 (PMC5633143; doi:10.1371/journal.pone.0184798)
Supplement: S1 Table — (DOC) [file pone.0184798.s001.doc]

**Chemometrics analysis of serum, urine and tissue samples.**

PLS discriminant models were built based on the ^1^H NMR data. Firstly, the ability of the models to discriminate between the tested groups was evaluated (through assessing all the serum, urine and tissue variables). The ROC curves and the AUC values obtained from this procedure for each model are presented in S1 Table.

**S1 Table.** The parameters of PLS-DA models obtained from ^1^H NMR analysis of serum, urine, tissue and fusion data samples.

| Comparison | Body fluid | AUC | *P* value | Q^2^(cum) | Number of latent variables |
| --- | --- | --- | --- | --- | --- |
| BDG vs. RG | SERUM | 0.97 | 0.220 | 0.376 | 2 |
| RG vs. UDG |  | 0.51 | 1.000 | N/A | 2 |
| BDG vs. UDG |  | 0.92 | 0.072 | 0.470 | 2 |
| BDG vs. RG | URINE | 0.85 | 0.511 | 0.230 | 2 |
| RG vs. UDG |  | 0.71 | 1.000 | N/A | 2 |
| BDG vs. UDG |  | 0.80 | 0.374 | 0.296 | 2 |
| BDG vs. RG | TISSUE | 0.94 | 0.140 | 0.446 | 2 |
| RG vs. UDG |  | 0.68 | 0.898 | 0.030 | 2 |
| BDG vs. UDG |  | 0.89 | 0.028 | 0.709 | 2 |
| BDG vs. RG | FUSION | 1.00 | 0.001 | 0.770 | 2 |
| RG vs. UDG |  | 0.74 | 0.380 | 0.366 | 2 |
| BDG vs. UDG |  | 1.00 | 0.01 | 0.589 | 2 |
