# Supplementary material for: The influence of different diets on metabolism and atherosclerosis processes—A porcine model: Blood serum, urine and tissues 1H NMR metabolomics targeted analysis
Source: PLoS One. 2017 Oct 9;12(10):e0184798. doi: 10.1371/journal.pone.0184798 (PMC5633143; doi:10.1371/journal.pone.0184798)
Supplement: S2 Table — (DOC) [file pone.0184798.s002.doc]

Because they were unsatisfactory, a selection of variables based on VIP plots with the jackknife confidence interval and 0.95 confidence level was applied for further analysis and to improve the model prediction. Subsequently, the selected metabolites for each block and comparison were used to construct new discriminant PLS models. Moreover, the new prediction models were assessed using the ROC curves and the AUC values (S2 Table, S1-S3 Figs).

**S2 Table.** The parameters of PLS-DA-VIP models obtained from ^1^H NMR analysis of serum, urine, tissue and fusion data samples.

| **Comparison** | **Body fluid** | **AUC** | ***P* value** | **Q^2^(cum)** | **Number of latent variables** |
| --- | --- | --- | --- | --- | --- |
| BDG vs. RG selected variables | SERUM | 1.00 | 0.018 | 0.634 | 2 |
| RG vs. UDG selected variables |  | 0.96 | 0.032 | 0.562 | 2 |
| BDG vs. UDG selected variables |  | 0.97 | 0.003 | 0.698 | 2 |
| BDG vs. RG selected variables | URINE | 0.87 | 0.933 | 0.240 | 2 |
| RG vs. UDG selected variables |  | 0.92 | 0.108 | 0.448 | 2 |
| BDG vs. UDG selected variables |  | 0.85 | 0.933 | 0.214 | 2 |
| BDG vs. RG selected variables | TISSUE | 0.99 | 0.381 | 0.555 | 2 |
| RG vs. UDG selected variables |  | 0.78 | 0.885 | 0.155 | 2 |
| BDG vs. UDG selected variables |  | 0.93 | 0.076 | 0.538 | 2 |
| BDG vs. RG selected variables | FUSION | 1.00 | 0.000 | 0.829 | 2 |
| RG vs. UDG selected variables |  | 1.00 | 0.000 | 0.863 | 2 |
| BDG vs. UDG selected variables |  | 1.00 | 0.001 | 0.783 | 2 |
