# Supplementary material for: The influence of different diets on metabolism and atherosclerosis processes—A porcine model: Blood serum, urine and tissues 1H NMR metabolomics targeted analysis
Source: PLoS One. 2017 Oct 9;12(10):e0184798. doi: 10.1371/journal.pone.0184798 (PMC5633143; doi:10.1371/journal.pone.0184798)
Supplement: S1 Fig — The VIP-PLS-DA models, ROC curve and AUC values obtained from serum samples based on selected variables according to the VIP plots with the jackknife confidence interval: A, B, C (BDG vs. RG); D, E, F (RG vs. UDG); G, H, I (BDG vs. UDG). Red diamonds—balanced diet group (BDG); blue boxes—regression group (RG); yellow pentagons—unbalanced diet group (UDG). (DOC) [file pone.0184798.s003.doc]

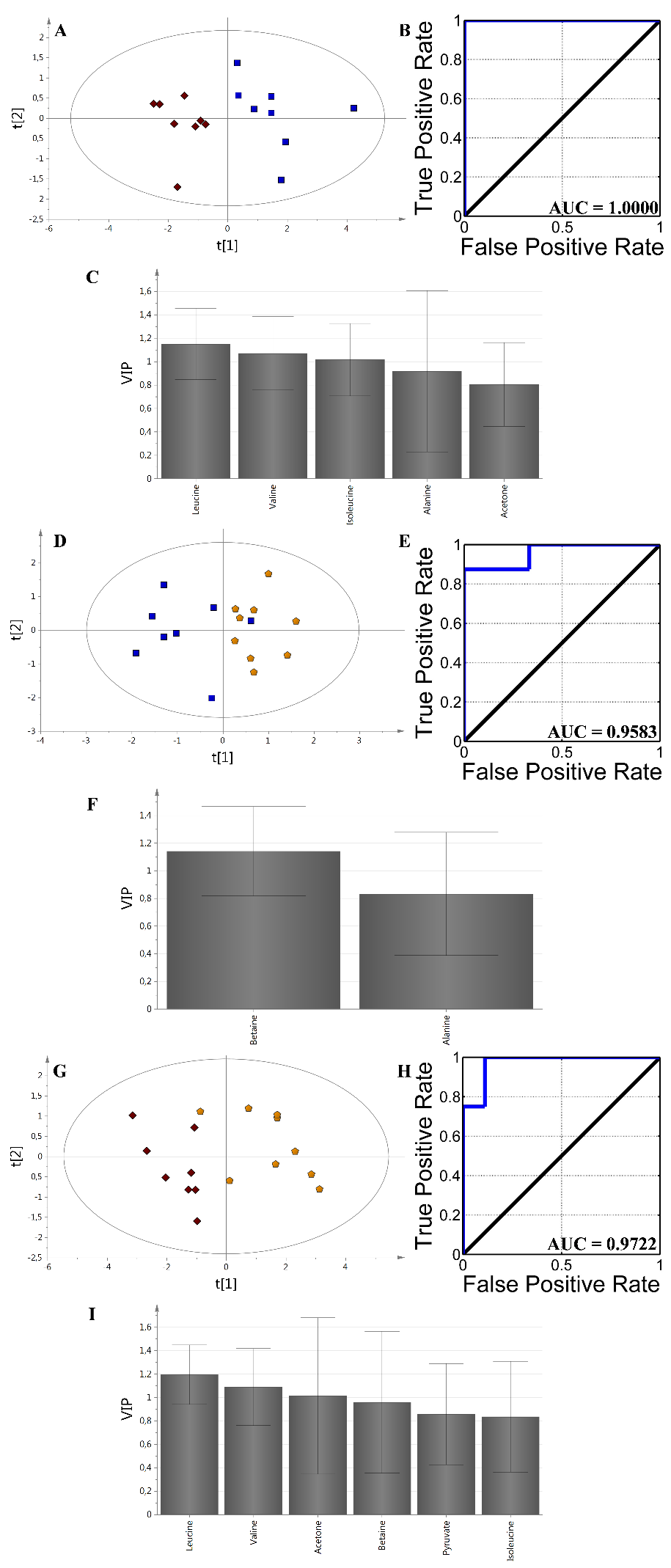


**S1 Fig.** The VIP-PLS-DA models, ROC curve and AUC values obtained from serum samples based on selected variables according to the VIP plots with the jackknife confidence interval: A, B, C (BDG vs. RG); D, E, F (RG vs. UDG); G, H, I (BDG vs. UDG). Red diamonds - balanced diet group (BDG); blue boxes - regression group (RG); yellow pentagons - unbalanced diet group (UDG).
