# Supplementary material for: The influence of different diets on metabolism and atherosclerosis processes—A porcine model: Blood serum, urine and tissues 1H NMR metabolomics targeted analysis
Source: PLoS One. 2017 Oct 9;12(10):e0184798. doi: 10.1371/journal.pone.0184798 (PMC5633143; doi:10.1371/journal.pone.0184798)
Supplement: S1 Text — (DOCX) [file pone.0184798.s006.docx]

**S1 Text. Information of the diet ingredients.**

**Regular (control) diet for pigs**

**Amount (kg/1000kg) Ingredient**

388,6307 Wheat

350,0000 Triticale

100,0000 Sunflower meal

66,1206 Canola cake

50,0000 Barley

14,0824 NOVITOL-30 *

8,3625 L-lysine

6,6487 Chalk

5,6239 Dicalcium phosphate

3,0725 NaCl

2,0000 Neubacid Liquid (acidifier)

2,0000 890141 PRMX 0,2% GROWER T.CH.(vitamins)

1,3527 L-THREONINE

1,0000 Na_2_SO_4_

0,5000 AVEMIX MCT-OIL 6/8

0,2500 Choline chloride 75%

0,1561 RHODIMET 88%

0,1000 Natuphos 5000

0,0500 AROMEX ME 67 000, 0,01 0,0050

0,0500 Rovabio 8

**1000,0000 Total weight**

*NOVITOL-30 COMPOSITION: Each kg contains: Sorbitol, Methionine, Vitamin C, Vitamin E, Choline chloride, Inositol, Vitamin B12.

**COMPOSITION**

**Name Unit AMOUNT**

1 MASS KG 1,0000

2 TOTAL PROTEIN % 15,0659

3 ENERGY NETTO KCAL/KG 2 300,0000

4 RAW FAT % 3,5028

5 FIBER % 4,3456

6 DRY MASS % 86,6905

7 DRY ASH % 3,6173

8 SALT-NaCl % 0,4740

9 Sodium –Na % 0,1700

10 Calcium –Ca % 0,5500

11 Phosphorus total % 0,4721

12 Phosphorus absorbable % 0,2200

13 Lysine % 0,9450

14 Methionine % 0,2836

15 Methionine + Cystine % 0,6024

16 Threonine % 0,6310

17 Tryptophan % 0,1857

18 Arginine % 0,8690

19 Starch % 47,4836

20 Vitamine A IU/KG 10 000,0000

21 VIT. D3 IU/KG 2 000,0000

22 VIT. E MG/KG 77,4654

23 VIT. K3 – added MG/KG 1,5000

24 VIT. B1 –added MG/KG 2,0000

25 VIT. B2 –added MG/KG 5,0000

26 Nicotinic acid. - added MG/KG 20,0000

27 Panthotenic ac. added MG/KG 11,9999

28 VIT.B6 added MG/KG 4,0000

29 VIT. B12 - added MCG/KG 25,0000

30 Biotin added MCG/KG 70,0000

31 Choline chloride added MG/KG 187,5000

32 Folic acid added MG/KG 0,7000

33 Potassium K % 0,4540

34 Chlorine % 0,3057

35 Ferrum MG/KG 167,7265

36 Ferrum added MG/KG 80,0000

37Copper Cu MG/KG 28,1502

**Atherogenic diet for pigs**

**Amount (kg/1000kg) Ingredient Percent**

300,000 Barley 30,00

220,0000 Triticale 22,00

200,0000 Concentrate. 20% FINISZER 20,00

140,0000 Tallow (beef) 14,00

100,0000 Wheat bran 10,00

40,0000 Sugar 4,00

**1000,0000 Total weight 100,00**

**COMPOSITION**

**Name Unit AMOUNT**

1 MASS KG 1,0000

2 TOTAL PROTEIN % 13,0

3 ENERGY NETTO KCAL/KG 2588,12

4 RAW FAT % 16,0

5 FIBER % 4,33

6 DRY MASS % 85,68

7 DRY ASH % 4,6

8 SALT-NaCl % 0,44

9 Sodium –Na % 0,1700

10 Calcium –Ca % 0,6900

11 Phosphorus total % 0,45

12 Phosphorus absorbable % 0,21

13 Lysine % 0,81

14 Methionine % 0,24

15 Methionine + Cystine % 0,4

16 Threonine % 0,57

17 Tryptophan % 0,155

18 Arginine % 0,67

19 Starch % 31,0

20 Vitamine A IU/KG 7500,0000

21 VIT. D3 IU/KG 1500,0000

22 VIT. E MG/KG 68,4654

23 VIT. K3 – added MG/KG 1,125

24 VIT. B1 –added MG/KG 1,5

25 VIT. B2 –added MG/KG 3,75

26 Nicotinic acid. - added MG/KG 15,0000

27 Panthotenic ac. added MG/KG 9,00

28 VIT.B6 added MG/KG 3,0000

29 VIT. B12 - added MCG/KG 18,75

30 Biotin added MCG/KG 52,5000

31 Choline MG/KG 987,5000

32 Potassium K % 0,5540

34 Chlorine % 0,39

35 Ferrum MG/KG 151,03

36 Ferrum added MG/KG 60,0000

37Copper Cu MG/KG 21,7
